# *OncoSolidDB*: An Oncology-Focused Curated Database of Ligand–Target Interactions for Precision Medicine Across Major Solid Cancers

**DOI:** 10.3390/cancers18101559

**Published:** 2026-05-12

**Authors:** Oussema Khamessi, Rihab Mahjoub, Ghada Mahjoub, Kais Ghedira

**Affiliations:** 1 Laboratory of Bioinformatics, Biomathematics and Biostatistics (BIMS-LAB) (LR16IPT09), Institut Pasteur de Tunis, University of Tunis El Manar, 13 Place Pasteur, BP74, Tunis 1002, Tunisia; rihab.mahjoub@insat.ucar.tn (R.M.); ghada.mahjoub@insat.ucar.tn (G.M.); 2 Higher Institute of Biotechnology Sidi Thabet ISBST, BiotechPôle, BP-66, University of Manouba, Ariana 2020, Tunisia

**Keywords:** solid tumor ligands, oncology drug database, ligand–receptor interactions, SMILES, bioinformatics, cancer database

## Abstract

**Background/Objectives:** The rapid expansion of targeted therapies has reshaped oncology by exploiting ligand-receptor interactions (LRI) to improve treatment specificity and patient outcomes. However, the data describing these ligands remain fragmented across multiple sources, limiting accessibility for researchers and clinicians. To address this gap, we developed the OncoSolidDB, the first curated and oncology-focused bioinformatics database dedicated to ligands associated with solid malignancies. **Methods:** OncoSolidDB integrates and harmonizes data from reliable repositories, including ChEMBL, DrugBank and the Anti-Cancer Fund, consolidating curated structural, chemical, pharmacological, and clinical annotations along with standardized identifiers. **Results:** The database currently encompasses 243 ligands across 15 major solid tumor types including breast, lung, colorectal, melanoma, prostate, gastric, ovarian, cervical, bladder, esophageal, head and neck, thyroid, pancreatic, renal and liver cancer (Hepatocellular Carcinoma, HCC). Each entry is annotated by standardized identifiers (DrugBank, ChEMBL), approval year, chemical structures (SMILES strings, 2D images), and downloadable protein structure files (PDB format). Temporal coverage spans 1953–2025, enabling exploration of historical trends in oncology drug approvals. The database content is suitable for bioinformatics analysis, molecular docking, virtual screening, ligand-based modeling, and drug repurposing studies. Outputs are available through a freely accessible web interface that supports search browsing by cancer type. **Conclusions:** By consolidating oncology-specific ligand data into a single, structured platform, OncoSolidDB offers a valuable resource for advancing drug discovery, repurposing strategies, and the rational design of next-generation targeted therapies for solid tumors. OncoSolidDB is accessible via our Bioinformatics Research PortalEinstein.

## Simple Summary

Cancer treatments increasingly rely on drugs designed to bind specific molecular targets in tumors. However, information about these therapeutic ligands is scattered across multiple databases, making it difficult for researchers to access structured and oncology-focused data in a single place. To address this challenge, we developed OncoSolidDB, a curated and openly accessible database dedicated to ligands targeting solid tumors. The database integrates chemical structures, standardized identifiers, pharmacological annotations, approval history and downloadable protein structural files. Covering 243 ligands across 15 major solid cancer types, OncoSolidDB enables researchers to explore molecular properties, perform computational modeling and support drug repurposing studies. By organizing oncology-specific ligand data into a harmonized platform, this resource aims to facilitate rational drug design, accelerate translational research and improve data accessibility for the scientific community.

## 1. Introduction

Cancer remains one of the leading causes of morbidity and mortality worldwide, with the global burden continuing to rise at an alarming rate. According to the latest estimates from the World Health Organization and the Global Cancer Observatory (GLOBOCAN), approximately 20 million new cancer cases and 9.7 million cancer-related deaths were recorded in 2022, with projections indicating that these numbers will reach nearly 35 million new cases annually by 2050 [1,2]. Among all cancer types, solid tumors, including breast, lung, colorectal, prostate, and melanoma, account for the majority of cases, representing over 90% of all malignancies and contributing substantially to global clinical and socioeconomic burdens. Regionally, the burden is particularly pronounced in North Africa and the Middle East, where cancer incidence is expected to double by 2040, driven by population growth, aging and lifestyle transitions [3,4]. Despite significant advances in oncology, including the development of chemotherapies, targeted therapies, and immunotherapies, many solid tumors continue to exhibit intrinsic or acquired drug resistance, profound tumor heterogeneity and off-target toxicities that limit long-term efficacy and patient outcomes [5,6].

Bioinformatics has emerged as a key discipline for addressing these challenges by enabling the study of ligand–receptor interactions at the molecular level. Small-molecule ligands, peptides, or biologics that bind to cellular receptors play a central role in modulating oncogenic pathways and form the backbone of precision oncology. Understanding their chemical diversity, structural features, and interaction profiles is critical for rational drug discovery, drug repurposing, and the design of next-generation therapies. However, such efforts require reliable, comprehensive, and oncology-specific datasets that integrate chemical, pharmacological, and structural information in a user-friendly and accessible manner. Several public chemical and pharmacological databases provide valuable information for cancer drug research, including DrugBank [7], ChEMBL [8,9,10], and the Therapeutic Target Database (TTD) [11].

While these repositories offer rich annotations on drug molecules, protein targets and bioactivity data, they are designed as general-purpose resources rather than oncology-specialized platforms. Consequently, cancer-related ligands are often mixed with non-cancer compounds, making it difficult to extract oncology-specific datasets systematically. Moreover, structural bioinformatics features such as standardized protein ligand complex files (in PDB format), curated SMILES strings, and cancer-type specific drug associations are often incomplete, scattered or missing [12,13].

This lack of oncology-focused integration hinders researchers who need streamlined access to curated ligand datasets for structural modeling, virtual screening, or comparative pharmacology studies. To address these gaps, we developed OncoSolidDB, a dedicated and integrative database focusing exclusively on ligands associated with solid tumors [14,15,16]. OncoSolidDB consolidates data from reliable repositories, including DrugBank, ChEMBL, and the Anti-Cancer Fund and harmonizes them into a cancer-specific framework. The database currently hosts 243 ligands covering 15 major solid tumor types, including breast, cervical, bladder, colorectal, esophageal, gastric, head and neck, liver (Hepatocellular Carcinoma), lung, melanoma, ovarian, pancreatic, prostate, renal and thyroid cancers. Each ligand entry is enriched with structural (SMILES, 2D images, and PDB files), chemical, pharmacological, and clinical annotations, as well as cross-references to major identifiers (DrugBank and ChEMBL). In addition, the database captures temporal approval data (1953–2025), enabling exploration of historical trends in oncology drug development. Uniquely, OncoSolidDB provides an interactive web-based interface that supports searching and browsing by cancer type, ligand annotation, and visualization of ligand overlaps across cancers, making the resource practical for both exploratory and hypothesis-driven research [17,18,19]. The overarching objective of this work is to present the solid tumor section of OncoSolidDB and demonstrate its potential as a specialized, oncology-focused ligand repository for structural bioinformatics and drug discovery applications.

To the best of our knowledge, OncoSolidDB represents the first curated and oncology-focused database that integrates chemical, structural, pharmacological, and clinical data specifically for ligands targeting solid tumors, providing a unified platform not available in general-purpose chemical repositories such as DrugBank or ChEMBL. By offering curated, cancer-specific ligand datasets, the database facilitates downstream computational workflows such as molecular docking, virtual screening, ligand-based modeling, and receptor-ligand network analysis [20,21,22]. Its oncology-specific orientation provides researchers with a more targeted resource than general-purpose chemical databases, thereby reducing time and effort in data preprocessing. Ultimately, OncoSolidDB, publicly available via https://liganddb.pythonanywhere.com/solid_cancer (accessed on 14 January 2026) and through our Bioinformatic Research Portal (public web server titled “Einstein,” http://196.203.66.81/, accessed on 14 January 2026) aims to accelerate the discovery of novel therapeutic strategies, support drug repurposing, and contribute to the rational design of ligands in the fight against solid tumors.

## 2. Materials and Methods

### 2.1. Data Collection and Source Integration

OncoSolidDB was developed through a systematic and comprehensive collection of ligands associated with solid tumors. The primary source of ligand information was the Anti-Cancer Fund (https://www.anticancerfund.org/) database [14], which provides data on anti-cancer agents and their clinical indications. Additional data were retrieved from DrugBank (https://go.drugbank.com/ v5.0) and ChEMBL (https://www.ebi.ac.uk/chembl/, accessed on 14 January 2026, v33) by querying cancer-specific indications, such as “breast cancer”, “lung cancer”, and “pancreatic cancer”.

Each ligand entry underwent rigorous manual curation, ensuring correct association with the corresponding cancer type, verification of availability in the DrugBank and ChEMBL databases, accurate chemical identifiers and SMILES strings, and the removal of duplicate entries and harmonization of identifiers across sources following established curation practices for chemical databases [23,24,25,26].

For each ligand, the following data were collected and standardized: ligand name, approval year, DrugBank and ChEMBL IDs, cancer type, SMILES representation, 2D chemical structure and downloadable 3D PDB files.

### 2.2. Selection Criteria

A rigorous selection process was implemented to guarantee that only high-confidence, oncology-relevant ligands were included in OncoSolidDB. The following inclusion and exclusion criteria were applied to all candidate compounds retrieved from the source databases.

#### 2.2.1. Inclusion Criteria for Ligand Selection

To ensure the reliability, consistency, and scientific relevance of the data integrated into OncoSolidDB, a set of stringent inclusion criteria was established for ligand selection. Only compounds meeting all predefined conditions were retained for further analysis and database integration. Specifically, ligands were required to be either approved therapeutic agents or investigational molecules associated with solid tumors, with at least one validated cancer indication reported in curated resources such as DrugBank, ChEMBL, and Anticancer Fund. Additionally, each compound had to possess a valid and standardized chemical structure representation, including a SMILES string, to enable structural normalization and computational analyses such as docking and QSAR modeling. Cross-referencing across databases was ensured through the use of unique identifiers (DrugBank ID and ChEMBL ID), guaranteeing data traceability and interoperability. Finally, only ligands linked to at least one of the fifteen predefined solid tumor types included in the scope of OncoSolidDB were considered, thereby maintaining the thematic coherence and applicability of the database.

#### 2.2.2. Exclusion Criteria

To maintain the quality, accuracy, and biological relevance of the dataset, specific exclusion criteria were applied during the curation process. Entries were discarded if they were exclusively associated with hematological malignancies rather than solid tumors, ensuring alignment with the database’s scope. Compounds lacking a valid or complete chemical structure were also excluded, as this prevented proper structural standardization and downstream computational analyses. In addition, duplicate entries across multiple databases that could not be reliably reconciled using standardized identifiers were discarded to avoid redundancy and data inconsistency. Molecules with ambiguous, conflicting, or unsupported cancer indications were not retained, thereby ensuring the reliability of therapeutic annotations. Furthermore, non-therapeutic compounds, including diagnostic agents, imaging probes, and unrelated chemical entities, were excluded from the dataset. Following the rigorous application of these criteria, the final curated dataset comprised 243 ligands associated with 15 major solid tumor types, providing a robust and high-confidence resource for subsequent analyses.

### 2.3. Data Curation and Standardization Pipeline

Raw data collected from multiple sources were subjected to a multi-step curation and standardization pipeline to ensure data quality, consistency, and interoperability. First, ligand entries were screened for relevance based on predefined inclusion and exclusion criteria. Subsequently, duplicate records originating from different databases were identified and resolved through identifier harmonization using DrugBank and ChEMBL IDs. Next, chemical information was standardized by verifying ligand names, correcting inconsistencies, and ensuring the presence of valid SMILES representations. Cross-referencing across databases was performed to enrich each entry with consistent annotations, including cancer type, approval status, and structural information. Quality control procedures included manual validation of cancer indications, verification of structural data integrity, and removal of incomplete or ambiguous entries. The curated dataset was further checked for consistency in formatting and annotation to facilitate downstream computational analyses. This systematic curation process ensured the generation of a high-quality, non-redundant, and analysis-ready dataset suitable for structural bioinformatics and drug-discovery applications. All curation steps were performed following standardized and reproducible procedures to ensure dataset reliability.

### 2.4. Workflow of Database Construction

**Figure 1:**
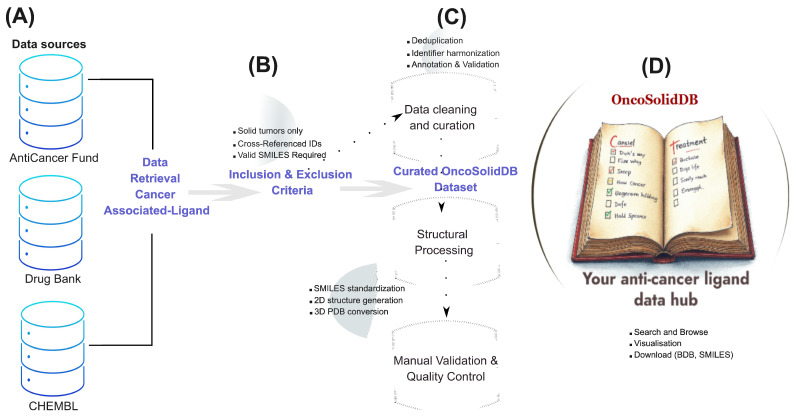
Schematic overview of the OncoSolidDB construction pipeline. The figure illustrates the multi-step workflow implemented to build the OncoSolidDB database, organized into four sequential panels. (**A**) Raw ligand data were retrieved from three primary repositories: DrugBank, ChEMBL, and the Anticancer Fund. Cancer-specific queries were performed using disease-related keywords to initially extract candidate ligands associated with solid tumor indications. (**B**) Retrieved ligands were subjected to stringent inclusion and exclusion criteria. Inclusion required that ligands be approved or investigational agents with validated solid tumor indications, valid chemical structures (SMILES strings), and cross-referenced identifiers (DrugBank and ChEMBL IDs). Exclusion criteria removed compounds associated exclusively with hematological malignancies, those lacking structural information, duplicate entries, molecules with ambiguous indications and non-therapeutic agents. (**C**) Following eligibility filtering, a multi-step curation pipeline was applied, including manual validation of cancer indications, structural standardization using RDKit and Open Babel, SMILES-to-PDB conversion, cross-database identifier harmonization, and quality control procedures to ensure data integrity and consistency. (**D**) The final curated and standardized ligand collection was integrated into a web-accessible platform, providing users with search, browse, and download functionalities, along with interactive visualizations, and structural data outputs. This workflow ensured the generation of a high-quality, non-redundant, and analysis-ready resource dedicated to ligands targeting solid tumors.

The overall workflow of OncoSolidDB construction is summarized in Figure 1. This schematic representation outlines the sequential steps of data acquisition, eligibility filtering, data curation and harmonization, structural processing, and integration into a web-accessible database. The workflow highlights the transformation of heterogeneous raw data into a curated, standardized, and computationally exploitable resource.

### 2.5. SMILES and Structural Processing

To ensure computational compatibility for structural bioinformatics applications, including molecular docking, virtual screening and molecular dynamics simulations, ligands were standardized into SMILES strings and converted into 2D images and 3D PDB files. SMILES extraction was performed using ChEMBL annotations [24], while structural generation was conducted using RDKit (v2023.09) and Open Babel (v3.1.1) [13,26,27]. The conversion from SMILES to PDB leveraged Open Babel’s interconversion capabilities, producing downloadable PDB files for each ligand. To facilitate reproducible ligand preparation, all workflows were executed using Python 3.11.5 within a Linux Ubuntu 22.04.4 LTS (Jammy) environment, ensuring robust handling of chemical structures, and integration into computational pipelines [27].

### 2.6. Web Interface Development

**Figure 2:**
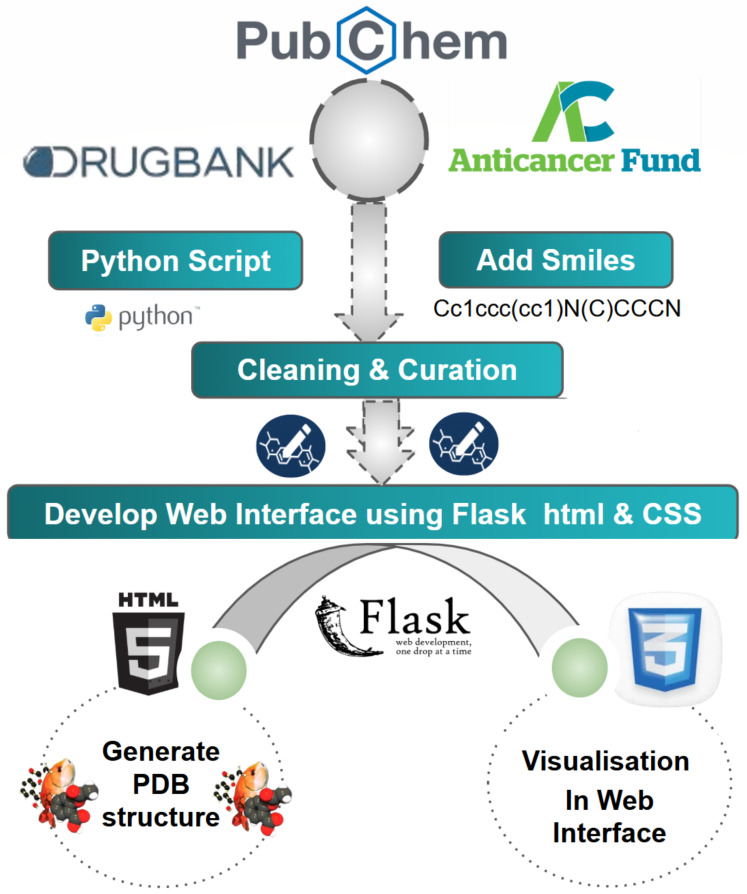
Technical architecture and implementation of the OncoSolidDB web platform. This figure illustrates the workflow integrating multiple data sources, including PubChem, DrugBank and the Anticancer Fund, into OncoSolidDB. Ligand data is processed using Python scripts for cleaning and curation, followed by the development of a web interface built with Flask, HTML5 and CSS3. The platform supports the generation of PDB structures and interactive visualization of chemical data within the web interface.

The OncoSolidDB web interface was developed to provide intuitive and interactive access to the curated dataset described in the construction workflow (Section 2.4). The platform translates processed data into a user-friendly environment for visualization, exploration and download. The overall technical architecture and implementation of the platform are illustrated in Figure 2.

The application was implemented using the Flask microframework (v2.3), which handles backend logic, routing, and dynamic data rendering. The frontend was developed using HTML5 and CSS3 to ensure a responsive and accessible user experience. The system architecture integrates curated ligand data into a structured database, enabling efficient querying based on cancer type, ligand name, or standardized identifiers such as DrugBank and ChEMBL IDs. Dynamic content delivery is achieved through template-based rendering, allowing seamless interaction between the database and the user interface. The web platform provides several core functionalities, including the ability to browse ligands by solid tumor category, visualize ligand annotations and metadata, display 2D chemical structures derived from SMILES strings, access these SMILES strings for computational workflows and download ligand structures in PDB format for more advanced use.

By decoupling data processing (Section 2.1, Section 2.2, Section 2.3, Section 2.4 and Section 2.5) from visualization, the web interface ensures efficient data access while maintaining scalability and usability for bioinformatics and drug-discovery applications.

Overview of the technical components used in the development of OncoSolidDB. The figure illustrates the integration of data-processing tools (Python, RDKit, Open Babel) with the web framework (Flask) and frontend technologies (HTML/CSS), enabling interactive visualization and access to curated ligand data [28].

### 2.7. Data Analysis and Visualization

To explore the characteristics and potential applications of the curated dataset, several analytical and visualization approaches were implemented. Descriptive statistical analyses were performed to assess the distribution of ligands across cancer types and approval years. These analyses were used to generate summary tables and histograms presented in the Section 3. Ligand overlap analysis across solid tumor types was conducted to identify compounds associated with multiple cancer indications. In this context, an “overlap” was defined as the presence of a single ligand annotated with more than one cancer type in the curated dataset. Based on this definition, a binary association matrix was constructed, linking ligands to their corresponding cancer types. This matrix was used to generate an UpSet plot, which provides a scalable alternative to Venn diagrams for visualizing intersections among multiple sets. The analysis highlights shared ligands across different cancer types, revealing potential opportunities for drug repurposing and multi-indication therapeutic strategies. All analyses and visualizations were performed using Python (version 3.11), with relevant libraries for data manipulation and visualization [29].

### 2.8. Availability and Access

OncoSolidDB is freely available for the scientific community through those links: https://liganddb.pythonanywhere.com/solid_cancer (accessed on 14 January 2026) and via our Bioinformatics Research Portal “Einstein” http://196.203.66.81/oncosoliddb/ (accessed on 14 January 2026).

## 3. Results

### 3.1. Database Content

**Figure 3:**
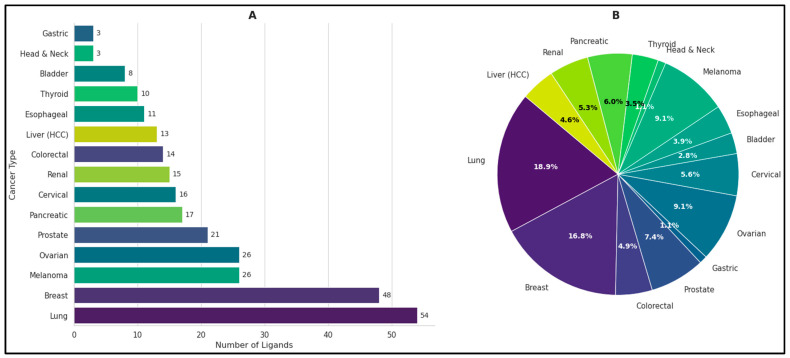
Distribution of ligands across solid tumor types. (**A**) Number of ligands associated with each solid tumor type in OncoSolidDB. Lung cancer and breast cancer represent the highest proportions, reflecting their high incidence and research focus. Intermediate representation is seen for ovarian cancer and melanoma, as well as prostate cancer and pancreatic cancer. Gastric and head and neck cancers are underrepresented, possibly due to fewer validated or structurally characterized ligands. (**B**) Relative proportions of ligands by cancer type, highlighting variability in dataset coverage across tumor types.

The solid tumor dataset of OncoSolidDB includes 243 ligands across 15 cancer types (Figure 3). The distribution of ligands reflects the relative research focus and clinical relevance of each solid tumor type, with lung and breast cancers accounting for the highest proportion of ligands. This distribution reflects both the intensity of research efforts and the clinical burden associated with different cancers. Notably, lung cancer (54 ligands, 18.9%) and breast cancer (48 ligands, 16.8%) account for the highest proportions, consistent with their high global incidence, extensive molecular characterization, and the availability of targeted therapies. Intermediate representation is observed for ovarian cancer and melanoma (26 ligands each, 9.1%), followed by prostate cancer (21 ligands, 7.4%) and pancreatic cancer (17 ligands, 6%) (Table S1).

These cancer types benefit from increasing interest in targeted treatment strategies and biomarker discovery. In contrast, cancers such as gastric cancer and head and neck cancer (3 ligands each, 1.1%) remain underrepresented, which may reflect limited availability of validated ligands–receptor data or fewer structurally characterized therapeutic compounds. Other cancer types, including cervical cancer (16 ligands, 5.6%), renal cancer (15 ligands, 5.3%), colorectal cancer (14 ligands, 4.9%), hepatitis-related cancers (13 ligands, 4.6%), esophageal cancer (11 ligands, 3.9%), thyroid cancer (10 ligands, 3.5%), and bladder cancer (8 ligands, 2.8%), show moderate representation within the dataset. This variability highlights disparities in research focus and therapeutic development across tumor types.

This curated dataset provides a comprehensive resource for researchers to explore ligand–receptor interactions, enabling structural bioinformatics analyses, virtual screening and drug-repurposing studies across a wide range of clinically important solid tumors [30].

### 3.2. Temporal Trends and Chemical Complexity

**Figure 4:**
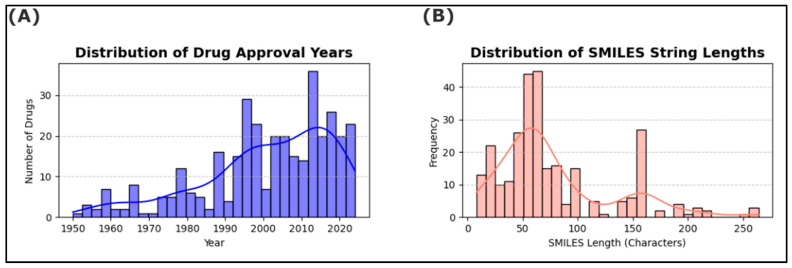
Temporal Trends and Chemical Complexity of Ligands in OncoSolidDB. (**A**) Histogram showing the distribution of ligand approval years in OncoSolidDB spanning 1953 to 2025. (**B**) Histogram of the SMILES string lengths illustrating the molecular complexity of ligands included in the database.

The distribution of ligand approval years in OncoSolidDB shows a heterogeneous temporal pattern across the period 1953–2025 (Figure 4A). Only a limited number of ligands are associated with approval dates prior to the late 1980s, corresponding to early anticancer agents. From the 1990s onward, a progressive increase in the number of approved ligands is observed, with a pronounced accumulation after the year 2000. The highest density of approvals occurred during the 2015–2025 period, indicating that a substantial proportion of ligands included in OncoSolidDB are associated with recent regulatory approvals.

Chemical complexity was assessed using the SMILES string length as a proxy for molecular size and structural elaboration (Figure 4B). The distribution reveals a broad range of SMILES lengths, spanning from short representations corresponding to small molecules to substantially longer strings associated with more complex compounds.

The majority of ligands exhibit SMILES lengths below 100 characters, with a median length of approximately 45 characters. A smaller subset of ligands displays markedly longer SMILES strings, exceeding 150 characters, reflecting increased molecular complexity within the dataset [31,32].

### 3.3. Ligand Overlap Across Solid Tumors

**Figure 5:**
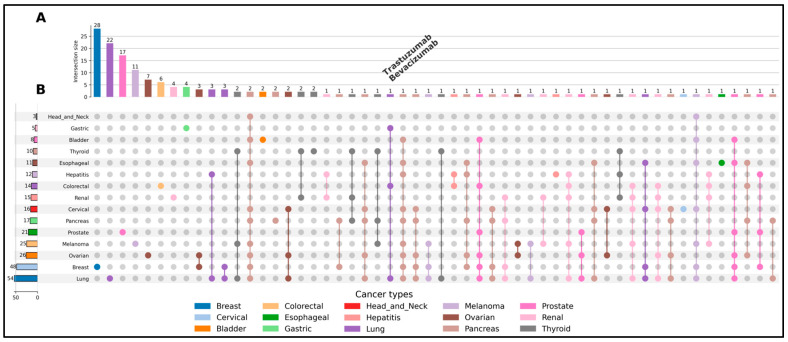
UpSet plot illustrating the distribution and overlap of ligands across 15 solid cancer types. The top panel (**A**) presents a bar chart where each vertical bar represents the size of an intersection, corresponding to the number of ligands shared among the specific combination of cancer types indicated in the matrix below. The bars are ordered in decreasing intersection size, with the tallest bars representing the most frequent intersection patterns. The bottom panel (**B**) displays a dot matrix in which each row corresponds to one of the 15 cancer types, labeled along the left side together with their total number of associated ligands. Each column represents a specific intersection. Filled colored dots indicate the cancer types involved in a given intersection and vertical connecting lines highlight ligands shared across multiple cancer types. Single filled dots without connecting lines correspond to ligands unique to a single cancer type.

An UpSet plot (Figure 5) was used to systematically analyze ligand sharing across 15 solid cancer types. The plot highlights both widely shared ligands and cancer-specific molecules, providing insights into therapeutic targeting strategies. For example, ligands such as trastuzumab and bevacizumab (Figure 5A) appear across multiple cancer types in the UpSet plot, including breast, lung, and colorectal cancers, illustrating their potential for drug repurposing. These molecules represent canonical examples of multi-cancer therapeutics, as they target fundamental oncogenic mechanisms rather than tissue-specific features. Trastuzumab specifically targets the HER2 receptor, a key driver of tumor proliferation in HER2-positive cancers [31], while bevacizumab inhibits VEGF-mediated angiogenesis, a process essential for tumor growth across many cancer types. Their presence in multiple intersections in the UpSet plot reflects their broad applicability and highlights their importance in drug-repurposing strategies [32]. Conversely, the plot also reveals ligands that are restricted to a single cancer type (Figure 5B), as indicated by the isolated dots (single intersections). These cancer-specific ligands may correspond to unique molecular alterations or tissue-specific pathways, making them promising candidates for precision medicine. For instance, certain ligands appear exclusively in cancers such as pancreatic or thyroid cancer, suggesting specialized roles in those tumor environments. Additionally, intermediate intersections involving two to four cancer types (e.g., shared ligands between lung and breast cancers or between ovarian and cervical cancers) suggest the presence of compounds targeting partially overlapping molecular pathways.

In contrast, certain ligands are uniquely associated with specific cancer types, such as gastric, head and neck, or thyroid cancers, indicating highly specialized therapeutic roles and potential relevance for precision medicine. Each intersection corresponds to a specific combination of cancer types, defined by the pattern of connected dots in Figure 5B, while the corresponding bar in Figure 5A represents the number of ligands associated with that combination. Overall, this analysis demonstrates that while some ligands act on universal cancer mechanisms, others exhibit high specificity, underscoring the importance of integrating both broad-spectrum and targeted therapeutic approaches in oncology.

The analysis reveals that the majority of ligands in OncoSolidDB are cancer type-specific, as evidenced by the predominance of single-dot intersections. This observation highlights the strong molecular specificity underlying targeted therapies in precision oncology. Nevertheless, a subset of ligands is shared across multiple tumor types, reflecting common oncogenic mechanisms.

### 3.4. Target Family Distribution

Functional classification revealed that kinase-associated ligands represent the dominant category within OncoSolidDB, reflecting the central role of kinase dysregulation in solid tumor pathogenesis. Receptor tyrosine kinases and intracellular signaling enzymes constitute the majority of annotated targets, followed by nuclear hormone receptors and proteasome-associated proteins. This distribution highlights the strong therapeutic focus on signaling-pathway modulation in precision oncology. Several ligands in OncoSolidDB are specifically kinase-targeting molecules, including inhibitors of EGFR, VEGFR, HER2, BRAF, and CDK family kinases. These ligands represent the dominant pharmacological category in the database, reflecting the central role of kinase dysregulation in solid tumor pathogenesis.

### 3.5. Temporal Approval Trends

Temporal analysis demonstrated a marked increase in solid tumor-targeting ligands following the year 2000, corresponding to the emergence of targeted therapies. A second acceleration phase was observed after 2010, coinciding with advancements in molecular diagnostics and personalized oncology. These findings underscore the growing complexity and specificity of anticancer drug development.

### 3.6. Database Overview and Interrogation

**Figure 6:**
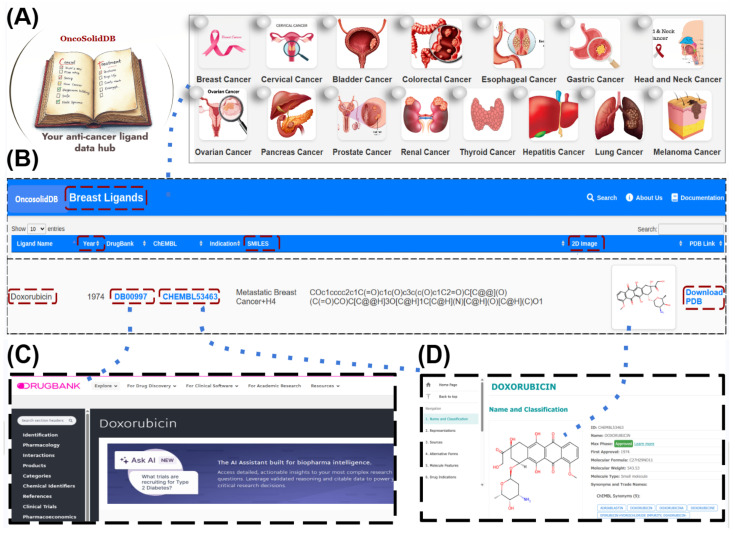
Overview of the OncoSolidDB web interface. The figure highlights the main database pages, including cancer-type browsing, ligand detail views, and structural data visualization. (**A**) browsing ligands by cancer type, (**B**) viewing ligand 2D chemical structures, (**C**) accessing DrugBank identifiers, and (**D**) accessing ChEMBL identifiers. Dashed arrows indicate the trend direction. Users can explore ligand-specific data, download 3D PDB structures and navigate between global overviews and detailed molecular-level information.

OncoSolidDB provides an interactive web-based interface that enables systematic interrogation of ligands associated with solid tumors (Figure 6). Users can explore the database through multiple entry points, including browsing by cancer type (Figure 6A) and searching by ligand name or standardized identifiers.

For each ligand, the database displays curated information such as cancer indication, approval year, DrugBank (Figure 6C), ChEMBL (Figure 6D) identifiers, SMILES representation, and two-dimensional (2D) chemical structure, as represented in Figure 6B. In addition, downloadable three-dimensional (3D) ligand structures in PDB format are provided to support structural bioinformatics analyses.

The database interface includes dedicated pages for cancer-specific ligand listings, individual ligand detail views, and summary visualizations illustrating ligand distribution across tumor types https://liganddb.pythonanywhere.com/cancer_type/breast or http://196.203.66.81/oncosoliddb/cancer_type/breast (accessed on 14 January 2026). These features allow users to navigate between global overviews and detailed molecular-level information, facilitating efficient retrieval and inspection of solid tumor–associated ligands [33,34].

## 4. Discussion

OncoSolidDB provides a specialized, oncology-focused view on ligands targeting solid tumors, addressing a gap left by general-purpose chemical databases. Unlike broader repositories such as DrugBank and ChEMBL, which cover a wide range of bioactive molecules across multiple therapeutic areas, OncoSolidDB contextualizes ligands by cancer type, enabling translational and computational oncology research [35,36]. This specificity is particularly valuable in oncology, where therapeutic efficacy depends on tumor biology, molecular targets, and clinical context [37,38]. In this way, OncoSolidDB complements but does not duplicate the scope of larger repositories. Furthermore, OncoSolidDB addresses the limitations of current oncology resources by bridging the gap between general drug-target mapping and tumor-specific application. While the Anticancer Fund focuses on clinical outcomes and canSAR on drug discovery informatics, OncoSolidDB provides a specialized focus on solid tumors, a level of detail often missing in broader repositories like the Therapeutic Target Database [39]. The development of OncoSolidDB addresses a critical gap in oncology bioinformatics by centralizing ligand–target information specific to solid malignancies. While several general drug repositories exist, none are specifically curated for solid tumor-focused ligand exploration with integrated structural and regulatory annotations [40]. In its current version, OncoSolidDB comprises 243 curated ligands associated with 15 major solid tumor types. While this number may appear limited compared to large-scale chemical repositories, the dataset was intentionally curated to prioritize data quality, structural consistency, and cross-database validation. Only well-characterized ligands with reliable annotations and complete structural information were included, ensuring high confidence in downstream computational applications. The database architecture has been designed to support continuous updates, and future releases will incorporate additional investigational compounds, ligands in clinical trials and newly approved therapeutics, thereby progressively expanding the dataset. The predominance of kinase-targeting ligands within the database reflects the central role of aberrant signaling pathways in tumor progression [41]. The observed temporal acceleration in drug approvals aligns with the paradigm shift toward molecularly targeted therapies and precision oncology strategies. Beyond data aggregation, OncoSolidDB provides a structured framework suitable for computational modeling, network pharmacology and AI-driven drug repurposing.

By enabling standardized ligand retrieval and cross-cancer comparison, this platform may facilitate hypothesis generation and translational research. In addition to data curation, preliminary analytical approaches were implemented to explore the structure and utility of the dataset. For instance, ligand overlap analysis across cancer types enables the identification of compounds associated with multiple tumor indications, highlighting potential opportunities for drug repurposing. Although the current analyses remain primarily descriptive, they demonstrate the potential of OncoSolidDB as a foundation for more advanced computational studies, including network pharmacology, predictive modeling, and integrative multi-omics analyses. Future expansions may include integration of genomic mutation profiles, resistance-associated variants, and multi-omics annotations to further enhance its precision oncology applications. An important extension of the database will involve the integration of corresponding target proteins and ligand–protein interaction data. Future developments will incorporate protein annotations from resources such as UniProt and the Protein Data Bank (PDB), including experimentally resolved ligand–protein complexes when available. In cases where structural data are lacking, predicted protein models may be integrated to support structure-based analyses [42]. This extension will enable a more comprehensive representation of ligand–target interactions and further enhance the utility of OncoSolidDB for structural bioinformatics and drug discovery applications. Similar domain-specific bioinformatics initiatives, such as DisintegrinDB (http://196.203.66.81/, accessed on 14 January 2026), have demonstrated the value of curated, disease- or molecule-focused databases in accelerating biomedical discovery [43]. While OncoSolidDB is primarily a curated bioinformatics resource, its cancer-type-specific ligand annotations can facilitate the design of targeted therapies and support precision medicine initiatives by highlighting potential drug candidates for specific tumor profiles [44,45].

While several large chemical and pharmacological databases exist, including DrugBank, ChEMBL and the Anticancer Fund, these resources were designed as general-purpose repositories rather than oncology-specific platforms. In these databases, cancer-related ligands are typically embedded within very large collections of bioactive molecules covering diverse therapeutic areas, meaning that extracting a curated dataset specifically associated with solid tumors requires extensive filtering and manual preprocessing. OncoSolidDB addresses this limitation by providing a domain-specific database dedicated exclusively to ligands targeting solid tumors, distinguished by several key features: a cancer-type specific organization enabling rapid identification of ligands associated with particular tumor types; integrated structural information including SMILES representations, 2D chemical structures and downloadable 3D PDB files; curated cross-references linking DrugBank and ChEMBL identifiers and data formats directly usable for structural bioinformatics workflows such as molecular docking; virtual screening; and molecular dynamics simulations. To our knowledge, no existing resource simultaneously provides curated ligand annotations, structural representations (SMILES and PDB), and cancer-type associations within a single unified platform specifically focused on solid tumors.

## 5. Conclusions

OncoSolidDB consolidates curated data on ligands targeting solid tumors into a single, freely accessible resource. Although the current release includes a focused set of 243 ligands, emphasis was placed on data quality, curation rigor and structural completeness to ensure reliability for computational applications. By combining structural, chemical and pharmacological annotations with interactive visualization, it bridges the gap between bench science and computational oncology.

Looking ahead, several perspectives are envisioned to extend the scope and impact of OncoSolidDB. These include the expansion of the dataset through the integration of additional investigational compounds, clinical trial candidates, and newly approved therapeutics, as well as the incorporation of ligand–target interaction data and protein structural information. Future developments will also focus on generating AI-ready datasets to support the application of high-performance computing HPC approaches in drug discovery [46], machine learning and deep learning approaches for predicting ligand–receptor interactions, optimizing drug design and advancing precision oncology [47,48,49]. Collectively, these future developments will strengthen OncoSolidDB as a dynamic and evolving resource, ultimately empowering the cancer research community in the pursuit of more effective targeted therapies. Furthermore, the integration of analytical and visualization approaches demonstrates the potential of the database not only as a curated repository but also as a resource for generating biologically and pharmacologically relevant insights. OncoSolidDB provides a structured platform not only for computational analyses but also to guide precision oncology research, supporting rational drug design and potential patient-specific therapeutic strategies.

## Data Availability

The database is freely available at: https://liganddb.pythonanywhere.com/solid_cancer and http://196.203.66.81/oncosoliddb/ (accessed on 14 January 2026). All curated datasets used in this study are accessible through the web interface.

## Supplementary Materials

The following supporting information can be downloaded at: https://www.mdpi.com/article/10.3390/cancers18101559/s1, Table S1: Distribution of ligands across solid tumor types.

## Abbreviations

AI	Artificial Intelligence
ChEMBL	Chemical database of bioactive molecules with drug-like properties
CSS	Cascading Style Sheets
DDR1	Discoidin Domain Receptor 1
FAIR	Findable, Accessible, Interoperable, Reusable
HCC	Hepatocellular Carcinoma
HER2	Human Epidermal Growth Factor Receptor 2
HTML	HyperText Markup Language
HPC	High-performance computing
LRI	Ligand–Receptor Interactions
ML	Machine Learning
PDB	Protein Data Bank
QSAR	Quantitative Structure–Activity Relationship
SMILES	Simplified Molecular Input Line Entry System
TTD	Therapeutic Target Database
UniProt	Universal Protein Resource
VEGF	Vascular Endothelial Growth Factor
