# Supplementary material for: OncoSolidDB: An Oncology-Focused Curated Database of Ligand–Target Interactions for Precision Medicine Across Major Solid Cancers
Source: Cancers (Basel). 2026 May 12;18(10):1559. doi: 10.3390/cancers18101559 (PMC13204178; doi:10.3390/cancers18101559)
Supplement: Supplementary file 1 [file cancers-18-01559-s001.zip › cancers-4208022-supplementary.pdf]

Supplementary Data S1:

Table S1. Distribution of ligands across solid tumor types

Solid Tumor Type	Number of Ligands	Percentage
Lung Cancer	54	18.9%
Breast Cancer	48	16.8%
Colorectal Cancer	14	4.9%
Prostate Cancer	21	7.4%
Gastric Cancer	3	1.1%
Ovarian Cancer	26	9.1%
Cervical Cancer	16	5.6%
Bladder Cancer	8	2.8%
Esophageal Cancer	11	3.9%
Melanoma (Skin)	26	9.1%
Head & Neck Cancer	3	1.1%
Thyroid Cancer	10	3.5%
Pancreatic Cancer	17	6%
Renal Cancer	15	5.3%
Hepatitis-related	13	4.6%
